# Mechanical Properties and Chemical Durability of Nafion/Sulfonated Graphene Oxide/Cerium Oxide Composite Membranes for Fuel-Cell Applications

**DOI:** 10.3390/polym12061375

**Published:** 2020-06-18

**Authors:** Dong Chan Seo, Ikseong Jeon, Eun Suk Jeong, Jae Young Jho

**Affiliations:** School of Chemical and Biological Engineering, Seoul National University, Seoul 08826, Korea; dongchan@snu.ac.kr (D.C.S.); backam777@snu.ac.kr (I.J.); esjeong@snu.ac.kr (E.S.J.)

**Keywords:** Nafion, sulfonated graphene oxide, cerium oxide, chemical durability

## Abstract

To improve both the mechanical and chemical durability of Nafion membranes for polymer electrolyte membrane fuel-cells (PEMFCs), Nafion composite membranes containing sulfonated graphene oxide (SGO) and cerium oxide (CeO_2_; ceria) were prepared by solution casting. The structure and chemical composition of SGO were investigated by FT-IR and XPS. The effect of the sulfonation, addition of SGO and ceria on the mechanical properties, proton conductivity, and chemical stability were evaluated. The addition of SGO gave rise to an increase in the number of sulfonic acid groups in Nafion, resulting in a higher tensile strength and proton conductivity compared to that of graphene oxide (GO). Although the addition of ceria was found to decrease the tensile strength and proton conductivity, Nafion/SGO/ceria composite membranes exhibited a higher tensile strength and proton conductivity than recast Nafion. Measurement of the weight loss and SEM observations of the composite membranes after immersing in Fenton’s reagent indicate an excellent radical scavenging ability of ceria under radical degradation conditions.

## 1. Introduction

Polymer electrolyte membrane fuel-cells (PEMFCs) are attracting attention as a clean and efficient source of energy due to their advantages of a high energy density, low emission of pollutants, and low corrosion. Nafion, a perfluorosulfonic acid polymer, is widely used as a polymer electrolyte membrane because of its high proton conductivity [1,2]. However, the deterioration of Nafion due to stress-buildup as well as chemical degradation was frequently observed in fuel-cells in long-term operation [3,4,5]. As the major causes that determine the durability of Nafion are its mechanical property and chemical stability, enhancing them is highly desirable for applications as a fuel-cell membrane

Various organic or inorganic fillers, used as reinforcement materials, have been widely applied to enhance the mechanical properties of Nafion [6,7,8,9]. The incorporation of multi-walled carbon nanotubes (MWCNTs) into Nafion resulted in an increase in tensile strength, but due to the high electrical conductivity of the MWCNTs, it caused an electronic crossover which is detrimental to a fuel-cell membrane. The addition of inorganic fillers such as zirconium phosphate and titania into Nafion also showed an increased mechanical property under fully humidified conditions, but its performance as a fuel-cell membrane was not satisfactory due to the reduction in proton conductivity. In order to effectively improve the performance of the Nafion, it should be considered to enhance the mechanical properties without losing other properties.

The incorporation of graphene oxide (GO), which is in the form of a bulk two-dimensional solid having a strong covalent bond, is a method frequently used to enhance the mechanical properties of Nafion owing to its high stiffness. It has been reported that GO can significantly increase the modulus of Nafion, even if a small amount is used [10,11]. However, in some morphological studies, the inhomogeneous distribution of GO was observed in the Nafion matrix [12]. Further functionalization of GO with sulfonic acid groups could be a good way to solve this problem. Because Nafion is composed of a hydrophobic polytetrafluoroethylene (PTFE) backbone and hydrophilic sulfonic acid side chain, the functionalization of GO with sulfonic acid groups could improve the compatibility with Nafion. Furthermore, it is known that the addition of a sulfonic acid group causes a more effective proton transfer in Nafion [13]. This means that the incorporation of sulfonated GO (SGO) into Nafion could enhance the proton conductivity as well as the mechanical property.

The chemical degradation of a polymer electrolyte membrane is initiated by the formation of hydrogen peroxide generated by the electrochemical oxygen reduction during fuel-cell operation. Hydroxyl (•OH) and hydroperoxyl (•OOH) radicals are formed by the reaction of hydrogen peroxide with metal impurities, and they are considered to be the most important factor of degradation because they can chemically attack the polymer membrane [14]. To ensure the chemical stability of Nafion, a multilayer Nafion membrane with aromatic polymers have been reported [15,16,17]. Although they have improved the chemical stability of Nafion, it was inevitably accompanied by a decrease in proton conductivity due to the inherently low conductivity of the aromatic polymers.

As another approach to improve the chemical stability of Nafion, the incorporation of a radical scavenger has been proposed to capture reactive oxygen radicals [18,19,20]. One of the radical scavengers, ceria, has been shown to have an excellent free radical scavenging ability in biological systems. In the Nafion with a small amount of ceria, the degradation was mitigated and an extended lifetime of membranes was observed [21].

Efforts have been made to improve the performance of Nafion using SGO or ceria, but no studies have been reported to improve both its mechanical and chemical durability by the incorporation of all of them. In this regard, we propose a new Nafion composite membrane with both SGO and ceria. The proposed design is expected to enhance the mechanical property of Nafion composites as well as the chemical stability without loss of proton conductivity. Through the measurement of mechanical properties and chemical stability, we investigated the effect of SGO and ceria on the durability of Nafion, and optimized their content.

## 2. Materials and Methods

### 2.1. Materials

The Nafion solution (5 wt%) in aqueous alcohol and graphite powder used were commercial products of DuPont (Wilmington, DE, USA), with the trade name of D521, and Asbury Carbons with the trade name of 3775, respectively. Ceria, with a particle size of 25 nm or less, was purchased from Sigma-Aldrich (St. Louis, MO, USA). Sodium nitrate (NaNO_3_, ≥99%), potassium permanganate (KMnO_4_, ≥99%), sodium hydride (NaH, 90%), 1,3-propane sultone (98%), N,N-dimethylacetamide (DMAc, ≥99.5%), and iron(II) chloride tetrahydrate (FeCl_2_•4H_2_O, ≥99%) were also purchased from Sigma-Aldrich (St. Louis, MO, USA). Sulfuric acid (H_2_SO_4_, 95%), hydrochloric acid (HCl, 35%), tetrahydrofuran anhydrous (THF, 99%), and hydrogen peroxide (H_2_O_2_, 30%) were purchased from Daejung Chemicals (Siheung, Gyeonggi, Korea). All reagents were used as received without further purifications.

### 2.2. Preparation of Sulfonated Graphene Oxide (SGO)

GO was synthesized from graphite powder by the modified Hummer’s method [22], and SGO was prepared according to the following procedure [23]. GO (0.2 g) and NaH (2.0 g) were added slowly to 250 mL of anhydrous THF in sequence, and the mixture was stirred at 60 °C for 6 h. Added to the mixture was 1,3-propane sultone (2.0 g), and it was stirred at 80 °C for 24 h with constant stirring. The filtered product was immersed in 3 wt% aq. HCl for 12 h, and was washed with ethanol several times to remove the residuals. The product was dried in a vacuum oven at 80 °C for 24 h.

### 2.3. Preparation of Nafion Composite Membranes

The Nafion solution was first dried at 60 °C to remove the water and solvent. The obtained 320 mg of Nafion resin was dissolved in 10 mL of the DMAc, and was sonicated for 1 h. SGO and ceria were added to the Nafion/DMAc solution with different filler contents, and the mixture was sonicated for 2 h. The Nafion film was prepared by casting on a petri dish in the amount that would give a film with a thickness of ca. 0.05 mm. The Nafion film was dried on a leveled hot-plate at 80 °C for 4 h and in a vacuum oven at 140 °C for 2 h. After being removed from the petri dish, the Nafion film was pretreated. It was boiled in a 5 wt% aq. H_2_O_2_ for 1 h, rinsed with distilled water for 1 h, and immersed in 0.5 M H_2_SO_4_ for 1 h to remove the organic and metallic impurities. After washing again with distilled water, the Nafion film was dried at 60 °C under vacuum.

The composite membranes were coded with letters and numbers. The letters N, G and S referred to Nafion, GO and SGO, respectively. The numbers after the letter G or S referred to the weight fraction of GO and SGO, respectively, and those after the hyphen referred to the content of ceria in phr. For example, NS4-3 denoted a Nafion composite membrane containing a 4 wt% of SGO and 3 phr of ceria.

### 2.4. Characterization and Tests

The structure of GO and SGO was characterized by using Fourier transform infrared spectroscopy (FT-IR). The FT-IR spectra of GO and SGO samples were recorded using an FT-IR spectrometer (Thermo Scientific, Nicolet 6700, Waltham, USA) in the range of 4000 to 650 cm^−1^. X-ray photoelectron spectroscopy (XPS, KRATOS, AXIS-His, Manchester, U.K.) was used to investigate the surface elemental composition and atomic configuration of both GO and SGO.

The mechanical properties of the membrane were measured using a universal testing machine (UTM, Lloyds Instruments, LR10K, Bognor Regis, U.K.) for the dog-bone specimens with a gage length of 7.62 mm, a width of 3.18 mm, and a thickness of 0.05 mm at a crosshead speed of 10 mm/min.

The proton conductivity of the membranes was obtained using an impedance analyzer (Zahner, IM6ex, Kansas, USA) with a voltage amplitude of 0.01 V. All samples were measured at 80 °C under the condition of 100% relative humidity (RH). The x-intercept of the impedance plots was taken to be the ionic resistance of the electrolyte. The in-plane conductivity (σ) was calculated based on the equation:

$$\sigma =\frac{L}{R\;W\;t}$$

where *L* is the distance between the voltage sense electrodes, and *R*, *W* and t are the resistance, width and thickness of the membrane, respectively.

The membrane durability was evaluated by Fenton’s test. The membrane samples were immersed in 3 wt% aq. H_2_O_2_ containing 4 ppm FeCl_2_ (Fenton’s reagent). The durability tests were carried out at 80 °C for 72 h. Fenton’s reagent was replaced by a fresh solution every 24 h, and the weight loss of the membrane degraded by Fenton’s reagent was measured 3 times every 24 h. The morphology of the membranes before and after Fenton’s test was investigated with a field emission scanning electron microscope (FE-SEM, JEOL, JSM-6701F, Seoul, Korea).

## 3. Results and Discussion

### 3.1. Structure of Sulfonated Graphene Oxide

**Figure 1:**
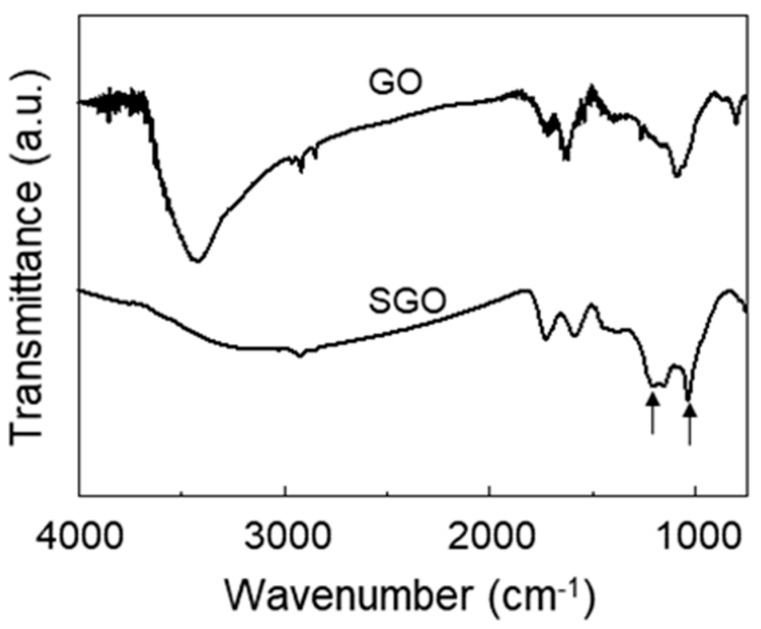
FT-IR spectra of GO and SGO.

To identify the introduction of a sulfonic group on the surface of the GO, we investigated the FT-IR spectra of GO and SGO, which are shown in Figure 1. In comparison with the spectrum of GO, the intensity of the O-H stretching peak at 3430 cm^−1^ was decreased in the spectrum of SGO, and new peaks due to the asymmetric and symmetric S=O stretching of sulfonic acid groups were observed at 1205 and 1030 cm^−1^, respectively [22,24]. This meant that the functional groups of GO were chemically modified through the sulfonation process.

**Figure 2:**
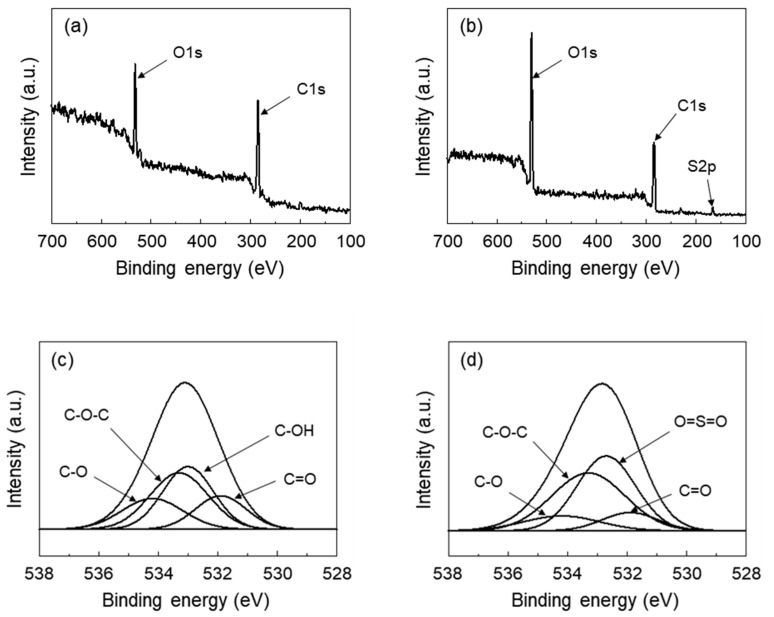
Wide region XPS spectra of (**a**) graphene oxide (GO) and (**b**) sulfonated graphene oxide (SGO). Deconvoluted XPS spectra in the O 1s region for (**c**) GO and (**d**) SGO.

The chemical composition of SGO in comparison with GO was analyzed by XPS as shown in Figure 2. In both the spectra of GO and SGO, the characteristic peaks attributed to the C 1s and O 1s components were observed at binding energies of 285.3 and 532.7 eV, respectively. Upon analysis of the XPS spectra shown in Figure 2b, a new peak appeared at 167.5 eV, corresponding to the S 2p binding energy of the sulfonic acid groups [22,23,24]. The spectra of GO and SGO were deconvoluted for O 1s to confirm clear evidence of the differences in chemical compositions, and it is shown in Figure 2c,d, respectively. The O 1s spectra of SGO were slightly different than those from GO. The C-OH peak at 533.0 eV disappeared, whereas a new peak at 532.7 eV corresponding to O=S=O bonds was observed [24]. From the peak area calculation, the GO and SGO areas of peaks corresponding to C-O-C at 533.3 eV were found to be 32.7% and 39.5%, respectively. The increase in intensity of the O=S=O and C-O-C bonds was attributed to the functionalization of GO through the sulfonation reaction. By the reaction between the propane sultone and the hydroxyl group of GO, the C-OH groups of GO were substituted with C-O-(CH_2_)_3_-SO_3_H groups. It was confirmed that the atomic percentage of SGO, as expected, was different from that of GO. The atomic percentages of O atoms for each sample were 30.8% and 36.0% for the GO and SGO, respectively. The atomic percentages of S atoms for the SGO also increased by 4.9%, compared to that of GO. These results indicated that the sulfonic acid groups were introduced on the surface of the GO as the hydroxyl groups of GO were replaced with the new functional groups.

### 3.2. Mechanical Properties

**Figure 3:**
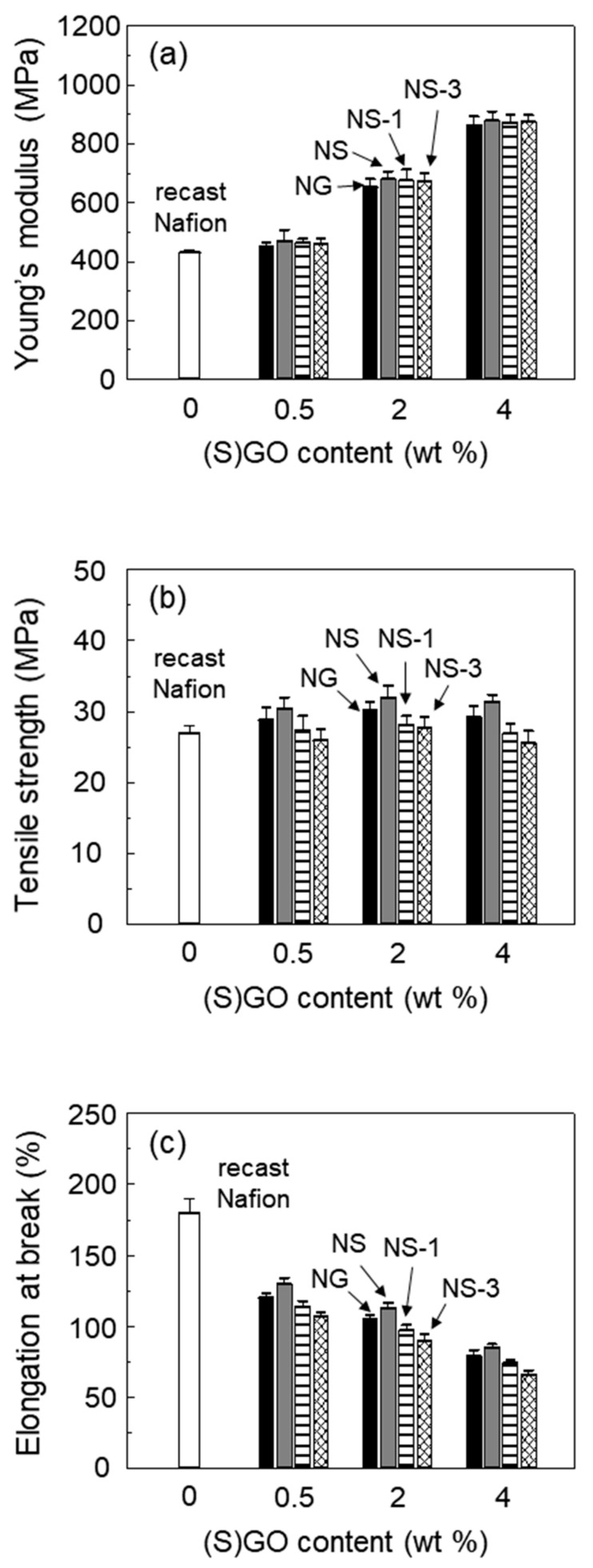
(**a**) Young’s modulus, (**b**) tensile strength and (**c**) elongation at break of recast Nafion and its composites with GO, SGO, and SGO/ceria in varying filler content.

The three tensile properties of recast Nafion and its composites are shown in Figure 3. The Young’s modulus of the Nafion composite membranes was increased with the increasing content of GO or SGO. Because the Young’s modulus is mainly influenced by the content of filler, the results were natural considering the incorporation of GO or SGO with a very high stiffness. The improvement of tensile strength by the incorporation of GO or SGO was not as great as the improvement of the Young’s modulus, because the elongation at break is typically decreased in composite with a weak interfacial adhesion between the rigid filler and matrix [25]. The tensile strength and elongation at break of the NS was slightly higher than that of the NG, which could be explained by a better interfacial adhesion of NS than that of NG [26]. As Nafion has sulfonic acid side chains, the dispersion of SGO in Nafion is better than that of GO. The tensile strength of the NG and NS tended to increase up to 2 wt% of the GO or SGO content, and decreased again at 4 wt% of the content. When the filler was incorporated in excess, it became aggregated, and could act as a stress concentrator at the interfaces [10].

On the other hand, the tensile strength slightly decreased as the content of ceria increased in the membranes. The lack of interfacial interaction between the ceria particles and the Nafion matrix decreased the elongation at break, resulting in a decrease in the tensile strength of the ceria-containing membrane. A similar result was reported for Nafion composites incorporating ceria [27]. Although the addition of ceria caused a decrease in the tensile strength of Nafion membranes, the Nafion/SGO/ceria composite membrane exhibited a higher tensile strength than recast Nafion.

### 3.3. Proton Conductivity

**Figure 4:**
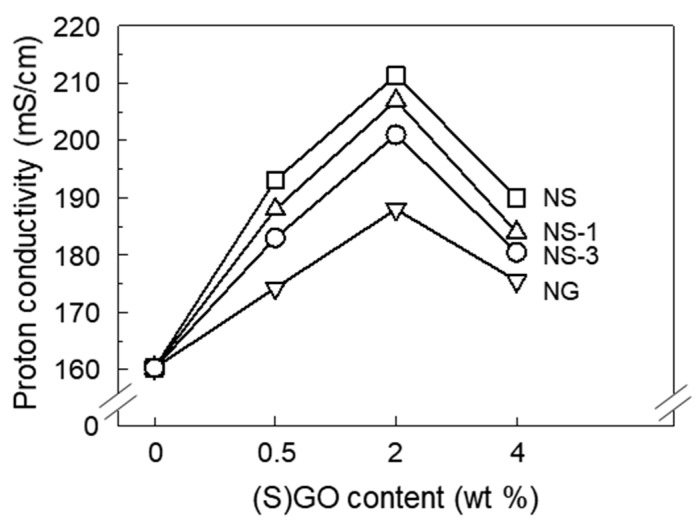
Proton conductivity of recast Nafion and its composites with GO, SGO, and SGO/ceria in varying filler content at 100% relative humidity (RH).

In order to investigate the effect of GO and SGO on the proton transfer properties of Nafion, the proton conductivities were calculated from Nyquist plot impendence measurements at 80 °C and 100% RH, as shown in Figure 4. The proton conductivity of the NG was higher than that of the recast Nafion. The transfer of protons in Nafion occurs in the presence of water molecules. In the presence of a water molecule, the sulfonic acid group in Nafion dissociates into a sulfonic acid anion and proton, and the water molecule carries a proton attached in the form of H_3_O^+^ along ion clusters composed of sulfonic acid groups [28]. The incorporation of hydrophilic GO under humid conditions allowed Nafion to absorb more water molecules, which could facilitate the transfer of protons (Figure S1 in the Supporting Information).

The degree to which the proton conductivity increased by the incorporation of SGO was greater than that of GO. The addition of sulfonic acid groups in Nafion due to the incorporation of SGO means that more transfer channels connected by an ion cluster were created, resulting in a more efficient proton transfer in the Nafion membrane [13,29]. The most effective proton transfer was achieved at 2 wt% of the SGO content. SGO, which is several micrometers in size, is known to block ion clusters of several nanometers, which are proton transport pathways [11]. Below 2 wt% of the SGO content, the effect of forming the proton transfer channel of the sulfonic acid group, by the incorporation of SGO, overwhelmed the blocking effect of SGO. As excess SGO was incorporated, the SGO began to agglomerate, and the blocking effect became larger so that the proton was not effectively transferred. The result of proton conductivity indicated that 2 wt% of the SGO content was the optimal amount for proton transfer in this study. As ceria was incorporated by the NS, the proton conductivity slightly decreased. It has been previously reported that the incorporation of non-proton-conductive material, such as ceria, into Nafion decreases proton conductivity [30].

### 3.4. Chemical Durability

Fenton’s reagent, which contains hydrogen peroxide (H_2_O_2_) and a small amount of Fe^2+^ ions, is mainly used for the chemical durability test of various kinds of polymer materials. The reaction of hydrogen peroxide with Fe^2+^ ions results in the formation of hydroxyl (•OH) and hydroperoxyl (•OOH) radicals. These radicals are known to attack the membrane and unzip the polymer backbone, leading to weight loss, thinning of the membrane, and the formation of pinholes [31,32].

**Figure 5:**
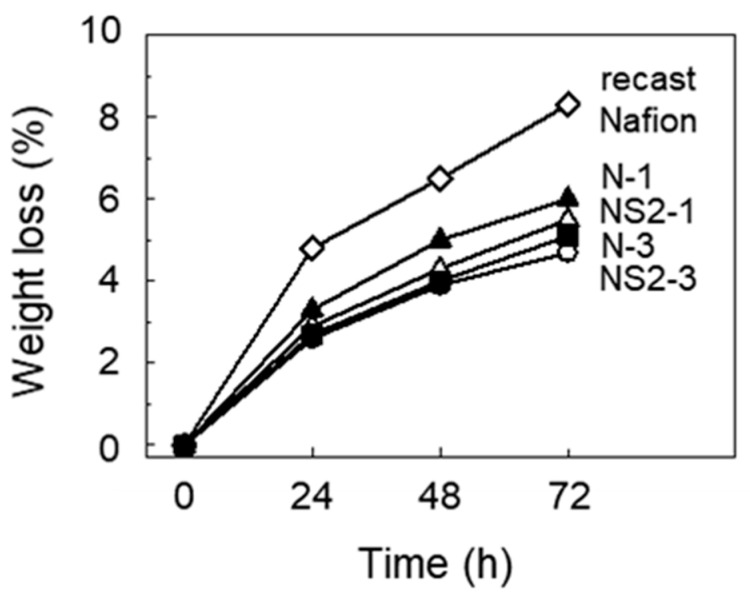
Weight loss of recast Nafion and its composites degraded by Fenton’s test.

Figure 5 shows the weight loss of each membrane after Fenton’s test. The weight loss of recast Nafion, N-1 and N-3, after immersion in Fenton’s reagent for 72 h was 8.3%, 6.0%, and 4.7%, respectively. It was therefore believed that ceria was efficient in improving the chemical durability of Nafion, considering that chemical degradation is accompanied by a reduction in the weight of the membrane. It is known that the undergoing of a reversible redox reaction between the Ce^3+^ and Ce^4+^ states of ceria causes the scavenging of radicals. The hydroxyl and hydroperoxyl radicals are neutralized by the oxidation of Ce^3+^ to Ce^4+^ and the reduction of Ce^4+^ to Ce^3+^, respectively [20,33]. The degree of weight loss was lower for the composite membranes with the incorporation of SGO at the same content of ceria, suggesting that the incorporation of SGO also improved the chemical durability. It has been reported that GO shows antioxidant activity in the form of hydroxyl radical scavenging [34].

**Figure 6:**
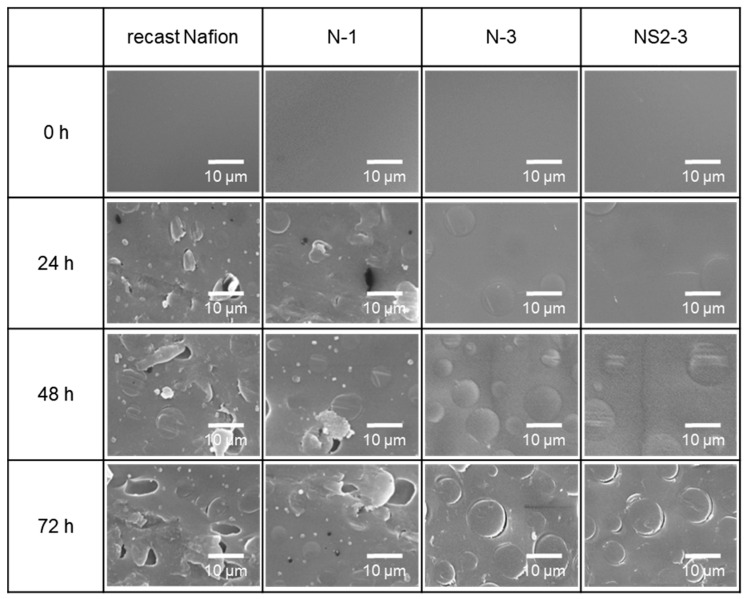
SEM images of recast Nafion and its composites after immersing in Fenton’s reagent for 0, 24, 48, 72 h.

Since the surface morphology might reflect the degree of degradation by Fenton’s reagent, the surface morphologies of recast Nafion and its composite membranes were examined. As shown in Figure 6, the surface of the recast Nafion became damaged after 24 h of reaction with Fenton’s reagent, and many pinholes were formed in the chemical degradation process as the reaction proceeded. This surface damage led to a significant weight loss of the recast Nafion, as can be seen in Figure 5. N-1 showed the same tendency, but the degree of damage and the number of pinholes were less than that of recast Nafion. Although small cracks appeared on the surface after a 72 h immersion in the Fenton reagent, no cracks or pinholes appeared on the surface of N-3 and NS2-3 after 48 h of immersion in Fenton’s reagent. The results above mean that the degree of chemical degradation of Nafion was reduced by the incorporation of ceria, which again corresponded to the results of weight loss by Fenton’s test.

## 4. Conclusions

Nafion composite membranes were prepared using SGO to improve mechanical and chemical durability. FT-IR and XPS spectroscopy indicated that the hydroxyl groups of GO were substituted with a new functional group containing a sulfonic acid group from the sulfonation reaction. With the addition of SGO, the Young’s modulus as well as the tensile strength of the Nafion composite membrane were enhanced. Although the incorporation of ceria interrupted the proton transfer in the Nafion membrane, the proton conductivity of the Nafion/SGO/ceria composite membranes was higher than the recast Nafion. It was due to the incorporation of SGO, which induced an increase in the number of sulfonic acid groups, and formed proton transport networks effectively in the Nafion membrane. The incorporation of both ceria and SGO resulted in less weight loss and surface damage during the chemical degradation process, indicating improved chemical durability. These results suggested that the Nafion/SGO/ceria composite membranes were suitable as a PEMFC with mechanical and chemical durability, and had excellent proton conductivity.

## Supplementary Materials

The following are available online at https://www.mdpi.com/2073-4360/12/6/1375/s1, Figure S1: (a) Water uptake and (b) swelling ratio of recast Nafion and its composites with GO, SGO, and SGO/ceria in varying filler content.
