# Supplementary material for: Mechanical Properties and Chemical Durability of Nafion/Sulfonated Graphene Oxide/Cerium Oxide Composite Membranes for Fuel-Cell Applications
Source: Polymers (Basel). 2020 Jun 18;12(6):1375. doi: 10.3390/polym12061375 (PMC7362198; doi:10.3390/polym12061375)
Supplement: Supplementary file 1 [file polymers-12-01375-s001.pdf]

Supporting Information

Mechanical properties and chemical durability of Nafion/Sulfonated graphene oxide/Cerium oxide composite membranes for fuel-cell applications

Dong Chan Seo, Ikseong Jeon, Eun Suk Jeong, and Jae Young Jho*

School of Chemical and Biological Engineering, Seoul National University, Seoul 08826, South Korea; dongchan@snu.ac.kr (D.C.S.); backam777@snu.ac.kr (I.J.); esjeong@snu.ac.kr (E.S.J.)

* Correspondence: jyjho@snu.ac.kr (J.Y.J.)

SI. Water uptake and swelling ratio

To investigate the effect of GO and SGO on water retention ability of Nafion composite membranes, water uptake (WU) and swelling ratio (SW) were measured, and these were determined by the change in weight and thickness between wet and dried membranes. The membrane samples with an area of 4 cm^{-1} were dried at 60 °C for 24 h. The weight and thickness of the dried samples were measured, and immersed distilled water at 80 °C for 24 h. After removing the water droplets on the surface, the weight and thickness were measured. The WU (%) and SW (%) were calculated form the following equation: WU (or SW) = 100 x $(X_{wet}-X_{dry})/X_{dry}$, where X is weight or thickness of mambrane samples.

The WU and SW of recast Nafion and its composites were shown in **Figure S1**. The WU and SW of the Nafion composite membranes were increased with increasing content of GO or SGO. It was due to the hydrophilic nature of GO.

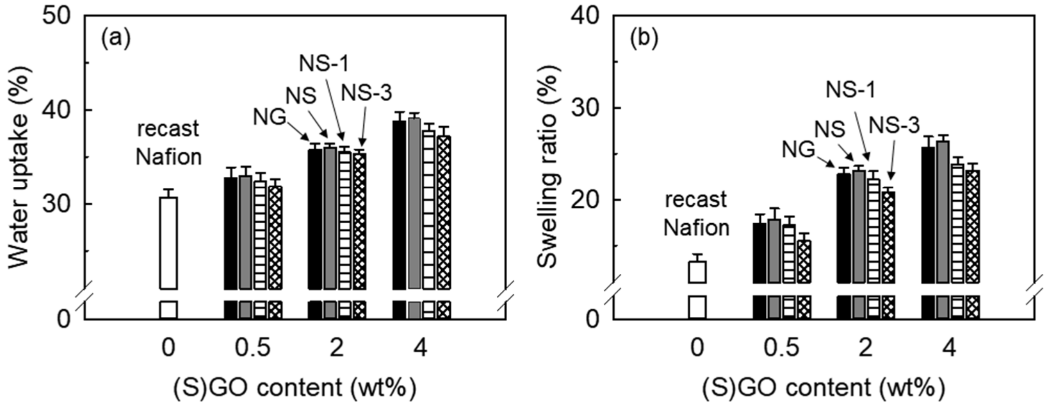

Figure S1. (a) Water uptake and (b) swelling ratio of recast Nafion and its composites with GO, SGO, and SGO/ceria in varying filler content.
